# Evaluating the Efficacy of Adipose-Derived Stromal Vascular Fraction Injection for Early-Stage Knee Osteoarthritis: A Multicenter Study

**DOI:** 10.3390/jcm15103855

**Published:** 2026-05-17

**Authors:** Aziz Atik, Ahmet Cemil Sökmen, Ercüment Zaim, Mert Emre Aydın

**Affiliations:** 1 Department of Orthopaedics and Traumatology, Faculty of Medicine, Balıkesir University, 10463 Balıkesir, Turkey; 2 Orthopaedics Clinic, Istanbul Silivri State Hospital, 34750 Istanbul, Turkey; 3 Orthopaedics Clinic, Istanbul Üsküdar State Hospital, 34662 Istanbul, Turkey; 4 Orthopaedics Clinic, Çan State Hospital, 17400 Çanakkale, Turkey; mertemre.aydin@saglik.gov.tr

**Keywords:** osteoarthritis, knee, adipose tissue, mesenchymal stem cells, stromal vascular fraction

## Abstract

**Background:** Knee osteoarthritis (KOA) is a major cause of disability worldwide, and adipose-derived stromal vascular fraction (SVF) has emerged as a potential regenerative treatment to modify disease progression. **Objective:** This study aimed to assess the effectiveness of autologous adipose-derived stromal vascular fraction (SVF) through intra-articular injection to treat early-stage knee osteoarthritis (KOA). **Materials and Methods:** This multicenter observational study (2019–2023) included adults aged 18–65 years with radiographically confirmed knee osteoarthritis. Patients were assigned to one of two groups through a retrospective, non-randomized process based on the actual treatment received during their clinical follow-up. Group T received intra-articular adipose-derived stromal vascular fraction (SVF) injections, while Group C received conservative treatment with non-steroidal anti-inflammatory drugs (NSAIDs) only. SVF was obtained from abdominal adipose tissue using a standardized closed-system device and injected intra-articularly. Pain and functional outcomes were assessed using the Visual Analog Scale (VAS) and Western Ontario and McMaster Universities Osteoarthritis Index (WOMAC) at baseline and at 1, 3, 6, 9, and 12 months. **Results:** Sixty-seven patients (41 SVF, 26 controls) were included, with comparable baseline characteristics (all *p* > 0.05). Preoperative VAS was lower in the SVF group (7.44 ± 1.44 vs. 8.31 ± 1.09; *p* = 0.029). At 12 months, VAS significantly decreased to 3.77 ± 1.49 in the SVF group, whereas it increased to 8.85 ± 0.67 in controls (*p* < 0.001). Similarly, baseline WOMAC scores were lower in the SVF group (62.6 ± 21.7 vs. 76.8 ± 8.76; *p* = 0.004). At 12 months, WOMAC improved to 29.4 ± 15 in the SVF group but worsened to 87.6 ± 3.21 in controls (*p* < 0.001). Within-group improvements were significant only in the SVF group (*p* < 0.001). No procedure-related complications were observed. **Conclusions:** The autologous adipose-derived stromal vascular fraction is an effective treatment option for early-stage KOA patients. However, a prospective, randomized, controlled study is warranted.

## Simple Summary

Knee osteoarthritis is a leading cause of disability worldwide, characterized by the painful breakdown of joint cartilage. Current treatments focus on managing pain rather than repairing the joint, creating a need for new regenerative options. This study investigated the effectiveness of a treatment called stromal vascular fraction (SVF), which is a mixture of healing cells and stem cells obtained from a patient’s own body fat. We compared patients who received an SVF injection into their knee to a control group who only used standard anti-inflammatory medications. Over 12 months, the SVF group experienced a significant and steady decrease in pain and a notable improvement in physical function. In contrast, patients using only medication saw their symptoms worsen over time. No serious side effects were reported, suggesting that SVF is a safe and effective way to help patients with early-stage osteoarthritis regain mobility and reduce pain. These findings are valuable to society as they offer a promising alternative that may slow disease progression and improve the quality of life for millions of people.

## 1. Introduction

Osteoarthritis (OA) affects more than 250 million people worldwide, and its global burden is expected to rise due to population aging, increased life expectancy, and growing obesity rates [1,2]. Recent data indicate that the global prevalence of knee osteoarthritis (KOA) is approximately 16.0%, with an annual incidence of 203 cases per 10,000 individuals [3]. The incidence of symptomatic KOA increases progressively with age and is more common in females than in males [4].

At present, there is no approved therapy capable of reversing age-related cartilage degeneration or subchondral bone deterioration. Therefore, the management of KOA remains predominantly conservative, aiming to relieve pain, preserve physical function, and slow structural disease progression.

Several intra-articular (IA) injection modalities have been proposed for the management of OA, including corticosteroids (CS), platelet-rich plasma (PRP), hyaluronic acid (HA), botulinum toxin type A, autologous conditioned serum (ACS), and stromal vascular fraction (SVF). Despite the extensive body of literature evaluating these interventions, determining the most effective strategy for symptomatic OA remains challenging [5,6,7,8]. SVF derived from adipose tissue, has emerged as a promising biologic therapy for knee osteoarthritis due to its rich cellular composition and regenerative potential [9]. Adipose tissue represents an abundant and easily accessible source of mesenchymal stem cells (MSCs), with higher isolation yields compared to other tissues [9]. SVF contains adipose-derived MSCs along with macrophages, pericytes, endothelial progenitor cells, and other stromal components, conferring immunomodulatory, anti-inflammatory, and pro-angiogenic properties [10]. Although its safety profile has been widely reported, clinical efficacy remains debated due to heterogeneity in preparation protocols and the limited number of high-quality studies incorporating validated structural outcome measures. Therefore, we aimed to evaluate the clinical efficacy and safety of intra-articular autologous adipose-derived stromal vascular fraction injection. Specifically, this study was designed to compare clinical outcomes between a retrospectively defined SVF treatment group and a control group receiving non-steroidal anti-inflammatory drugs (NSAIDs) through a non-randomized observational design.

## 2. Materials and Methods

### 2.1. Design

This multicenter observational study was conducted between 2019 and 2023. Patients were recruited from three centers. All eligible patients diagnosed and managed during the study period were consecutively included. Demographic characteristics, clinical findings, diagnostic evaluations, treatment details, and follow-up outcomes were retrospectively collected from institutional electronic medical records using a standardized data collection form. Data were anonymized prior to analysis. To minimize inter-center variability, uniform definitions and outcome measures were applied.

**Figure 1:**
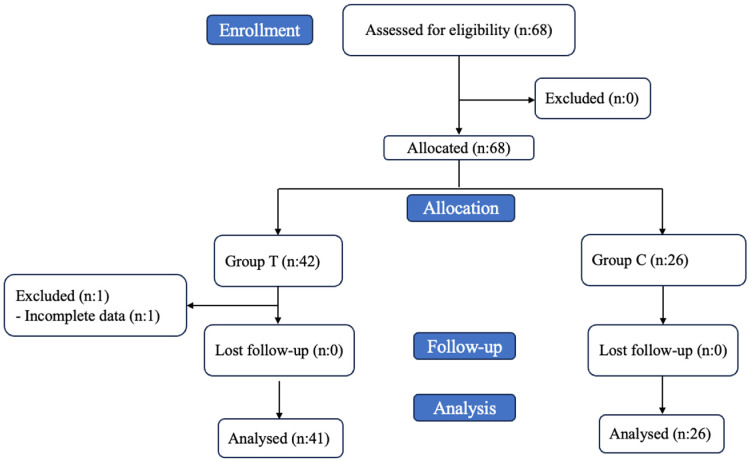
Flowchart of patient selection.

Patients were assigned to one of two groups through a retrospective, non-randomized process based on the actual treatment received during their clinical follow-up. Group T consisted of patients who underwent adipose-derived SVF injection. Group C served as the control group and received only non-steroidal anti-inflammatory drugs (NSAIDs) without SVF treatment. Patient assignment to either the SVF (Group T) or the control group (Group C) was based on a shared decision-making process in a real-world clinical setting. Eligible patients were informed about the potential benefits and procedural details of SVF injection as well as standard conservative options. The final assignment was determined primarily by patient preference and the clinical judgment of the treating physician. No formal randomization was performed. The patient selection and allocation process are given in the study flowchart (Figure 1).

### 2.2. Ethical Approval

This study was conducted in accordance with the ethical principles outlined in the Declaration of Helsinki. Ethical approval was obtained from the Institutional Clinical Research Ethics Committee (Approval No: 2019/117; 28 August 2019) prior to data collection. Due to the retrospective design of the study and the use of anonymized patient data, the requirement for informed consent was waived by the ethics committee.

### 2.3. Inclusion Criteria

Patients in the MSC group were eligible if they were between 18 and 65 years of age, had a body mass index (BMI) between 18.5 and 45 kg/m^2^, and were diagnosed with KOA classified as Kellgren–Lawrence (KL) Grade I or II, and in rare cases Grade III, based on radiographic evaluation.

### 2.4. Exclusion Criteria

Patients were excluded if they had inflammatory arthritis (including rheumatoid arthritis, psoriatic arthritis, or ankylosing spondylitis), sequelae of septic arthritis, a history of major knee trauma, or significant knee malalignment (defined as varus > 10° or valgus > 7°). Patients who had undergone any prior knee surgery, including arthroscopic procedures, were also excluded. Patients with ligament injuries (treated or untreated) before MSC administration were not eligible. In addition, those who had received conservative treatments such as physical therapy, rehabilitation modalities, or intra-articular injections (platelet-rich plasma, hyaluronic acid, or corticosteroids) within one year prior to MSC treatment were excluded. Patients with KL grade IV osteoarthritis were also excluded.

### 2.5. PICO Strategy

Population(s)/Problem(s): Adult patients aged 18–65 years with radiographically confirmed KOA, classified as KL Grade I–II (and selected Grade III cases).

Intervention(s): Intra-articular injection of adipose-derived SVF.

Control/Comparator: Conservative medical treatment consisting of non-steroidal anti-inflammatory drugs (NSAIDs) without SVF administration.

Outcome: The primary end point was a reduction in VAS and WOMAC scores.

### 2.6. Technique

All three participating orthopedic centers applied a standardized adipose tissue harvesting and processing protocol using the same closed-system device (Lipocell^®^, Meika Group, San Marino, Italy), a Class IIa medical device. Adipose tissue was harvested from the abdominal region under sterile conditions with the patient in the supine position. Following local anesthesia, a small stab incision was made in the right lower abdominal quadrant. Tumescent infiltration was performed using Klein solution (1 L normal saline, 50 mL lidocaine 1%, 1 mL epinephrine 1:1000, and 10 mL sodium bicarbonate 8.4%). Lipoaspiration was subsequently carried out to obtain adipose tissue. The harvested tissue was mechanically processed and filtered using the Lipocell^®^ system to obtain a MSC-rich SVF, which was then injected intra-articularly into the affected knee joint.

### 2.7. Data Collection

Demographic and clinical variables were systematically recorded for all patients, including age, sex, body mass index (BMI), comorbidities, radiographic osteoarthritis grade according to the Kellgren–Lawrence classification, injection side (right/left/bilateral knee), and the imaging modality used for diagnosis. Clinical outcomes were assessed using validated instruments. Pain severity was measured with the Visual Analog Scale (VAS), and functional status was evaluated using the Western Ontario and McMaster Universities Osteoarthritis Index (WOMAC). VAS and WOMAC scores were documented at baseline (pre-treatment) and at 1, 3, 6, 9, and 12 months of follow-up. The same evaluation schedule was applied to both the SVF group and the control group, which received conservative treatment without injection.

For patients in the SVF treatment group, procedural variables were additionally recorded, including the volume of adipose tissue harvested and the final processed adipose tissue yield obtained after mechanical processing. All data were retrospectively extracted from electronic medical records and analyzed according to the predefined study flowchart.

### 2.8. Statistical Analysis

Statistical analyses were performed using Jamovi (version 2.4.1; The jamovi project, Sydney, Australia). The distribution of continuous variables was assessed using the Shapiro–Wilk test. Continuous variables were presented as both mean ± standard deviation (SD) and as median (Q1, Q3). Categorical variables were expressed as frequencies and percentages. Between-group comparisons for continuous variables were performed using the independent samples *t*-test or the Mann–Whitney U test, as appropriate. Categorical variables were compared using the chi-square or Fisher exact test. Longitudinal intra-group comparisons of VAS and WOMAC scores were conducted using the Friedman test (repeated-measures non-parametric ANOVA). When overall significance was detected, post hoc pairwise comparisons were performed using the Durbin–Conover test. Mean differences (MDs) both in VAS and WOMAC scores were calculated for between-group comparisons at each time point. All tests were two-tailed, and a *p*-value < 0.05 was considered statistically significant.

## 3. Results

A total of 67 patients (51 females and 16 males) were included in the study, comprising 41 patients in Group T and 26 patients in Group C. The baseline demographic and clinical characteristics of the two groups are summarized in Table 1. The mean age was 54.05 ± 13.37 years in Group T and 59.4 ± 6.74 years in Group C. Although patients in Group C were numerically older, the difference did not reach statistical significance (*p* = 0.089). There was no statistically significant difference between groups in terms of gender distribution (*p* = 0.477). The difference in BMI between the two groups was not statistically significant (*p* = 0.122). Disease grade distribution was also comparable between groups (*p* = 0.554). In Group T, 22% (n = 9) of patients were Grade I, 70.7% (n = 29) were Grade II, and 7.3% (n = 3) were Grade III. In Group C, 11.5% (n = 3) were Grade I, 80.8% (n = 21) were Grade II, and 7.7% (n = 2) were Grade III. In two patients, injection was administered to the left knee only, and in one patient to the right knee only; all remaining patients received bilateral knee SVF injections.

Preoperative VAS scores were significantly lower in Group T compared to Group C (7.44 ± 1.44 vs. 8.31 ± 1.09; MD = 0.863; *p* = 0.029). At 1 month, Group T demonstrated significantly reduced pain levels (5.56 ± 1.94 versus 7.12 ± 1.39; MD = 1.554; *p* = 0.001). This difference increased over time. At 3 months, the mean VAS was 5.44 ± 1.95 in Group T and 8.04 ± 0.52 in Group C (MD = 2.599; *p* < 0.001). At 6 months, scores were 4.92 ± 2.25 versus 8.58 ± 0.50 (MD = 3.652; *p* < 0.001), and at 12 months, 3.77 ± 1.49 versus 8.85 ± 0.67 (MD = 5.071; *p* < 0.001). Within-group improvements were significant in Group T (*p* < 0.001) (Table 2).

**Figure 2:**
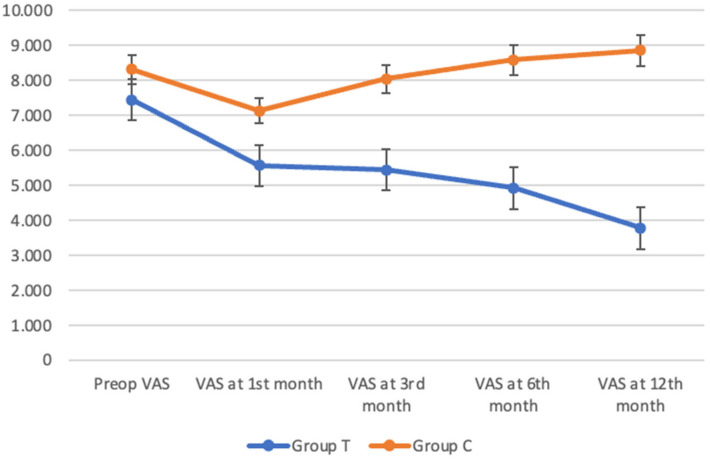
Longitudinal changes in VAS scores in Group T and Group C during 12-month follow-up.

While VAS scores in Group C did not demonstrate sustained improvement and progressively increased over time, Group T exhibited a consistent and gradual reduction in VAS scores at all follow-up time points, indicating continuous clinical improvement. The within-group decrease in VAS scores was statistically significant in Group T (*p* < 0.001). In contrast, although Group C showed a slight reduction at the first month compared to preoperative values, VAS scores increased in subsequent months (Figure 2). For intra-group comparisons, the Friedman test (repeated-measures non-parametric ANOVA) was applied. Following significant overall results, post hoc analysis was performed using the Durbin–Conover test for both groups separately. Detailed *p*-values for these comparisons are provided in Supplementary Tables S1 and S2.

Preoperative WOMAC scores were significantly lower in Group T compared to Group C (62.6 ± 21.7 versus 76.8 ± 8.76; MD = 14.2; *p* = 0.004). At 1 month, WOMAC scores decreased in Group T (48.5 ± 20.4), whereas Group C remained higher (72.7 ± 7.62; MD = 24.20; *p* < 0.001). The between-group difference widened over time. At 3 months, mean WOMAC scores were 46.5 ± 20.4 in Group T and 79.2 ± 5.7 in Group C (MD = 32.7; *p* < 0.001). At 6 and 12 months, scores were 37.9 ± 19.4 and 29.4 ± 15 in Group T versus 84.1 ± 5.11 and 87.6 ± 3.21 in Group C (*p* < 0.001). Within-group improvements were significant in Group T (*p* < 0.001) (Table 3).

**Figure 3:**
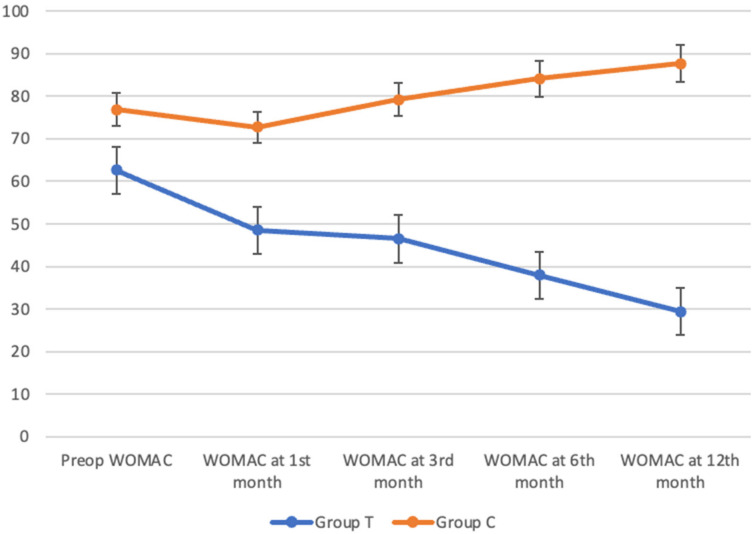
Longitudinal changes in WOMAC scores in Group T and Group C during 12-month follow-up.

While WOMAC scores in Group C did not demonstrate sustained improvement and progressively increased throughout the follow-up period, Group T showed a consistent and marked reduction in WOMAC scores at all time points, indicating continuous functional improvement. The within-group decrease in WOMAC scores was statistically significant in Group T (*p* < 0.001). In contrast, although Group C showed a slight reduction at the first month compared to baseline, WOMAC scores increased in subsequent months and exceeded preoperative levels (Figure 3). For intra-group comparisons, the Friedman test (repeated-measures non-parametric ANOVA) was applied. Following significant overall findings, post hoc pairwise comparisons were performed using the Durbin–Conover test separately for each group. Detailed *p*-values are presented in Supplementary Tables S3 and S4.

At the 12-month follow-up, the mean clinical improvement (delta) from baseline in the SVF group was 3.67 for VAS and 33.2 for WOMAC, whereas the control group exhibited a mean deterioration of −0.54 for VAS and −10.8 for WOMAC.

No intra-articular bleeding was observed in any patient during or after the procedure. Additionally, no hematoma, active bleeding, or ecchymosis was detected at the abdominal harvesting site. All procedures were performed on an outpatient basis, and all patients were discharged on the same day without immediate complications.

## 4. Discussion

This multicentric observation study revealed that intra-articular injections of autologous adipose-derived SVF injection was associated with significant within-group improvements. It is worth noting that patients who were injected with SVF experienced progressive decrease in VAS and WOMAC scores within one year of follow-up. On the contrary, no benefits were seen among patients from the control group, who also experienced worsening symptoms. No side effects were reported.

The primary aim of OA management is to halt or at least slow the progression of cartilage degeneration. SVF has emerged as a potential regenerative therapy with promising clinical implications. Previous studies have demonstrated that SVF provides both clinical and radiological improvement in patients with KOA [11]. Several investigations have specifically evaluated its efficacy in early-stage disease. In a recent study, Kim et al. reported that SVF offers functional benefits lasting approximately 6–12 months [12]. Contemporary biologic strategies in knee osteoarthritis management have shifted toward not only structural repair but also the modulation of the intra-articular environment [13]. The recent literature emphasizes the role of biological augmentation in cartilage preservation, where adipose-derived therapies like SVF act as immunomodulators that suppress pro-inflammatory cytokines, thereby influencing pain-related outcome assessments [13,14]. These advancements suggest that the efficacy of SVF may be linked to its ability to alter pain signaling pathways and enhance the regenerative niche of the subchondral bone and synovium [13,14]. Bolia et al. found that SVF yielded superior clinical outcomes compared to bone marrow aspirate, although radiological findings remained inconclusive [15]. Furthermore, Van Pham et al. demonstrated that the combination of SVF and platelet-rich plasma (PRP) promoted articular cartilage regeneration in a murine model [16].

Caforio et al. administered autologous SVF obtained from the abdominal region using the same commercial kit (Lipocell^®^) intra-articularly to 30 KOA patients who underwent arthroscopic lavage and debridement and were followed up for 1 year [17]. For clinical evaluations, the VAS and WOMAC scores were recorded prior to administration and at 1, 3, 6, and 12 months after administration. They reported that the mean pain score evaluated using the VAS decreased by 53% at 1 month and 83% at 1 year post-administration. Similarly, they reported a 47% improvement in WOMAC scores one month post-administration and an 84% improvement one year post-administration. The improvement rates reported in the VAS and WOMAC scores indicate a higher rate of improvement than those obtained in our study. This discrepancy may be attributed to administration of SVF in conjunction with arthroscopy. Additionally, in a prospective study, the ability to perform clinical applications and patient follow-ups in a more standardized manner would be more advantageous than that in a retrospective study, which may have contributed to a more favorable outcome [17].

The findings of the present study are consistent with previously published clinical studies evaluating intra-articular SVF therapy for knee osteoarthritis. Non-randomized controlled trials, such as those by Tran et al. showing enhanced outcomes when SVF was combined with microfracture, suggest a potential synergistic regenerative effect [18]. Cohort studies by Tsubosaka et al. and Yokota et al. further confirmed sustained improvements in pain, function, and imaging outcomes, reinforcing the clinical relevance of SVF across different patient populations and protocols [19,20]. Randomized controlled trials (RCTs) have supported these findings. RCTs such as those by Hong et al. and Garza et al. demonstrated that SVF provides significant improvements in pain and functional outcomes, particularly when compared with hyaluronic acid, other intra-articular treatments or a placebo [21,22]. In line with our results, most studies reported a favorable safety profile, with only minor and self-limiting adverse events, mostly related to the harvesting procedure rather than intra-articular injection. Case series, including those by Simunec et al. and Gibbs et al., also demonstrated improvements, particularly when SVF was combined with PRP, although the lack of control groups limits definitive conclusions [23,24]. Notably, while prior studies have generally reported clinical improvement beginning at 1–3 months, our findings indicate that symptom relief may occur earlier, as evidenced by significant improvements observed as early as the first month. Importantly, our findings suggest that intra-articular SVF may provide an earlier onset of clinical benefit than previously emphasized in the literature, as significant reductions in both VAS and WOMAC scores were observed as early as 1 month after treatment. In addition, these improvements were not transient but continued progressively throughout the 12-month follow-up, underscoring the potential of SVF as a safe and durable treatment option for early-stage knee osteoarthritis.

This study has several limitations that should be considered when interpreting the findings. First, the retrospective and non-randomized design introduces a potential risk of selection bias and limits the ability to establish a causal relationship between SVF treatment and clinical outcomes. Although baseline characteristics were statistically comparable, the presence of lower initial VAS and WOMAC scores in the SVF group may have influenced the observed treatment effect. Second, the relatively small sample size and the imbalance between groups may reduce the generalizability of the results and the statistical power of subgroup analyses. Third, the control group received only conservative NSAID therapy, without comparison to other commonly used intra-articular treatments such as hyaluronic acid or platelet-rich plasma, which limits the ability to position SVF within the current therapeutic hierarchy. Additionally, the absence of randomization and blinding may have introduced performance and assessment bias. The predominantly female cohort introduces potential sex bias, limiting generalizability, as biological responses to SVF may differ between males and females. Another important limitation is the lack of standardized quantification of SVF cellular content, which may contribute to variability in treatment efficacy. Additionally, the inclusion of a small KL Grade III subset introduces heterogeneity in disease severity. Finally, although clinical outcomes were comprehensively evaluated, the absence of advanced imaging or histological assessment limits the ability to draw conclusions regarding structural cartilage regeneration. However, the strength of this study is its multicenter design, which increases the generalizability of the findings, together with the statistical consistency of the results, as the SVF group demonstrated significant early, sustained, and progressively greater improvements in both VAS and WOMAC scores throughout follow-up. Future prospective, randomized controlled trials with larger cohorts and standardized protocols are needed to validate these findings.

## 5. Conclusions

This multicenter study demonstrates that intra-articular injection of autologous adipose-derived stromal vascular fraction (SVF) is a safe and highly effective intervention for patients with early-stage knee osteoarthritis. Our findings demonstrate significant and progressive improvements in both pain (VAS) and functional outcomes (WOMAC), with clinical benefits emerging as early as one month post-injection and remaining sustained throughout the 12-month follow-up period.

## Figures and Tables

**Table 1:** Baseline demographic and disease severity characteristics.

	Group T (n = 41)	Group C (n = 26)	*p*-Value
Age	54.05 ± 13.37	59.4 ± 6.74	0.089
Gender (F)	30 (73.2%)	21 (80.8%)	0.477
BMI	29.5 ± 4.07	31 ± 3.15	0.122
Grade			0.554
I	9 (22%)	3 (11.5%)	
II	29 (70.7%)	21 (80.8%)	
III	3 (7.3%)	2 (7.7%)	

F: female, BMI: body mass index, T: treatment with SVF, C: control.

**Table 2:** Changes in VAS scores over time.

		Group T (n = 41)	Group C (n = 26)	MD	*p*-Value
Preop VAS	Mean ± SD	7.44 ± 1.44	8.31 ± 1.087	0.863	0.029 *
	Median (Q1, Q3)	8 (6, 9)	8 (8, 9)		
VAS at 1st month	Mean ± SD	5.56 ± 1.94	7.12 ± 1.39	1.554	0.001 *
	Median (Q1, Q3)	6 (4, 7)	7 (7, 8)		
VAS at 3rd month	Mean ± SD	5.44 ± 1.95	8.04 ± 0.52	2.599	<0.001 *
	Median (Q1, Q3)	6 (5, 7)	8 (8, 8)		
VAS at 6th month	Mean ± SD	4.92 ± 2.25	8.58 ± 0.5	3.652	<0.001 *
	Median (Q1, Q3)	5 (3, 7)	9 (8, 9)		
VAS at 12th month	Mean ± SD	3.77 ± 1.49	8.85 ± 0.67	5.071	<0.001 *
	Median (Q1, Q3)	4 (3, 5)	9 (8, 9)		
*p*-value		<0.001 ^#^	<0.001 ^#^		

VAS: Visual Analog Scale, SD: standard deviation, T: treatment, C: control, MD: mean difference, Q1: Quartile 1 (25 percentile), Q3: Quartile 3 (75 percentile), *: Mann–Whitney U test, ^#^: Friedman test.

**Table 3:** Changes in WOMAC scores over time.

		Group T (n = 41)	Group C (n = 26)	MD	*p*-Value
Preop WOMAC	Mean ± SD	62.6 ± 21.7	76.8 ± 8.76	14.2	0.004 *
	Median (Q1, Q3)	61.5 (47.7, 81.8)	75.5 (70, 83.5)		
WOMAC at 1st month	Mean ± SD	48.5 ± 20.4	72.7 ± 7.62	24.2	<0.001 *
	Median (Q1, Q3)	51 (38.7, 58)	72.5 (68, 78)		
WOMAC at 3rd month	Mean ± SD	46.5 ± 20.4	79.2 ± 5.7	32.7	<0.001 *
	Median (Q1, Q3)	49.7 (38, 60.4)	80 (77, 83)		
WOMAC at 6th month	Mean ± SD	37.9 ± 19.4	84.1 ± 5.11	46.2	<0.001 *
	Median (Q1, Q3)	39.8 (25.3, 49.5)	85 (82, 87.8)		
WOMAC at 12th month	Mean ± SD	29.4 ± 15	87.6 ± 3.21	58.2	<0.001 *
	Median (Q1, Q3)	28 (18.2, 40.7)	88 (87, 89.8)		
*p*-value		<0.001 ^#^	<0.001 ^#^		

WOMAC: Western Ontario and McMaster Universities Osteoarthritis Index, SD: standard deviation, T: treatment, C: control, MD: mean difference, Q1: Quartile 1 (25 percentile), Q3: Quartile 3 (75 percentile), *: Mann–Whitney U test, ^#^: Friedman test.

## Data Availability

The data that support the findings of this study are openly available.

## Supplementary Materials

The following supporting information can be downloaded at: https://www.mdpi.com/article/10.3390/jcm15103855/s1, Table S1: Post hoc test of VAS scores for Group T; Table S2: Post hoc test of VAS scores for Group C; Table S3: Post hoc test of WOMAC scores for Group T; Table S4: Post hoc test of WOMAC scores for Group C.

## References

[1] Peshkova M., Lychagin A., Lipina M., Di Matteo B., Anzillotti G., Ronzoni F., Kosheleva N., Shpichka A., Royuk V., Fomin V. (2022). Gender-Related Aspects in Osteoarthritis Development and Progression: A Review. Int. J. Mol. Sci..

[2] Moldovan F., Gligor A., Moldovan L., Bataga T. (2023). An Investigation for Future Practice of Elective Hip and Knee Arthroplasties during COVID-19 in Romania. Medicina.

[3] Cui A., Li H., Wang D., Zhong J., Chen Y., Lu H. (2020). Global, regional prevalence, incidence and risk factors of knee osteoarthritis in population-based studies. eClinicalMedicine.

[4] Li D., Li S., Chen Q., Xie X. (2020). The Prevalence of Symptomatic Knee Osteoarthritis in Relation to Age, Sex, Area, Region, and Body Mass Index in China: A Systematic Review and Meta-Analysis. Front. Med..

[5] Cao K.X., Tang H.N.A., Tran M.H. (2026). Advances in hyaluronic acid therapy for knee osteoarthritis: Monotherapy and combination strategies: An evidenced based review. Orthop. Rev..

[6] Ruberto R.A., Dash A., Simmons S., Cooper H.J., Geller J.A., Herndon C.L. (2026). The Effects of Perioperative Intra-Articular Corticosteroids During Manipulation Under Anesthesia After Total Knee Arthroplasty: A Retrospective Study. J. Arthroplast.

[7] Riglet L., Delgado T., Lopez R.F., Pers Y.M., Cyteval C., Adam P., Ornetti P. (2026). Efficacy and safety of a combination of platelet-rich plasma with non-crosslinked hyaluronic acid versus a crosslinked hyaluronic acid, in single-injection for knee osteoarthritis. Randomized, controlled, multicenter, non-inferiority trial. BMC Musculoskelet. Disord..

[8] Lei B., Zhuang W., Wang Y., Ma S., Wang H., Zhang D., Han Q., Wang X., Ding Q., Li Y. (2025). Botulinum Toxin A (BTA) for pain and function improvement in osteoarthritis: A systematic review and meta-analysis of clinical efficacy evidence from randomized controlled trials. Toxicon.

[9] Kim Y.S., Suh D.S., Kwon Y.B., Chung J.H., Koh Y.G. (2026). Injection of Adipose-Derived Stromal Vascular Fraction Rapidly Relieves Pain in Patients with Knee Osteoarthritis. Medicina.

[10] Jeyaraman N., Shrivastava S., Rangarajan R.V., Nallakumarasamy A., Ramasubramanian S., Muthu S., Jeyaraman M. (2025). Comparative outcome analyses of stromal vascular fraction vs. nanofat in primary osteoarthritis knee: A double-blinded randomized controlled trial protocol. World J. Exp. Med..

[11] Aletto C., Oliva F., Maffulli N. (2022). Knee intra-articular administration of stromal vascular fraction obtained from adipose tissue: A systematic review. J. Clin. Orthop. Trauma.

[12] Kim K.I., Kim M.S., Kim J.H. (2023). Intra-articular Injection of Autologous Adipose-Derived Stem Cells or Stromal Vascular Fractions: Are They Effective for Patients with Knee Osteoarthritis? A Systematic Review with Meta-analysis of Randomized Controlled Trials. Am. J. Sports Med..

[13] Zhang K., Wang Z., He J., Lu L., Wang W., Yang A., Xie H., Huang L., Huang Y., Zhang K. (2025). Mechanisms of synovial macrophage polarization in osteoarthritis pathogenesis and their therapeutic implications. Front. Immunol..

[14] Hossain M.S., Lee H.J., Hossain R., Kim C.M., Lee C.J., Hwang S.C. (2026). Recent Advances in Therapeutic Approaches for Knee Osteoarthritis: A Narrative Review. Biomol. Ther..

[15] Bolia I.K., Bougioukli S., Hill W.J., Trasolini N.A., Petrigliano F.A., Lieberman J.R., Weber A.E. (2022). Clinical Efficacy of Bone Marrow Aspirate Concentrate Versus Stromal Vascular Fraction Injection in Patients with Knee Osteoarthritis: A Systematic Review and Meta-analysis. Am. J. Sports Med..

[16] Van Pham P., Hong-Thien Bui K., Quoc Ngo D., Tan Khuat L., Kim Phan N. (2013). Transplantation of Nonexpanded Adipose Stromal Vascular Fraction and Platelet-Rich Plasma for Articular Cartilage Injury Treatment in Mice Model. J. Med. Eng..

[17] Caforio M., Nobile C. (2021). Intra-Articular Administration of Autologous Purified Adipose Tissue Associated with Arthroscopy Ameliorates Knee Osteoarthritis Symptoms. J. Clin. Med..

[18] Tran T.D.X., Wu C.M., Dubey N.K., Deng Y.H., Su C.W., Pham T.T., Thi Le P.B., Sestili P., Deng W.P. (2019). Time- and Kellgren–Lawrence Grade-Dependent Changes in Intra-Articularly Transplanted Stromal Vascular Fraction in Osteoarthritic Patients. Cells.

[19] Tsubosaka M., Matsumoto T., Sobajima S., Matsushita T., Iwaguro H., Kuroda R. (2020). The influence of adipose-derived stromal vascular fraction cells on the treatment of knee osteoarthritis. BMC Musculoskelet. Disord..

[20] Yokota N., Hattori M., Ohtsuru T., Otsuji M., Lyman S., Shimomura K., Nakamura N. (2019). Comparative Clinical Outcomes After Intra-articular Injection With Adipose-Derived Cultured Stem Cells or Noncultured Stromal Vascular Fraction for the Treatment of Knee Osteoarthritis. Am. J. Sports Med..

[21] Garza J.R., Campbell R.E., Tjoumakaris F.P., Freedman K.B., Miller L.S., Santa Maria D., Tucker B.S. (2020). Clinical Efficacy of Intra-articular Mesenchymal Stromal Cells for the Treatment of Knee Osteoarthritis: A Double-Blinded Prospective Randomized Controlled Clinical Trial. Am. J. Sports Med..

[22] Hong Z., Chen J., Zhang S., Zhao C., Bi M., Chen X., Bi Q. (2019). Intra-articular injection of autologous adipose-derived stromal vascular fractions for knee osteoarthritis: A double-blind randomized self-controlled trial. Int. Orthop..

[23] Simunec D., Salari H., Meyer J. (2020). Treatment of Grade 3 and 4 Osteoarthritis with Intraoperatively Separated Adipose Tissue-Derived Stromal Vascular Fraction: A Comparative Case Series. Cells.

[24] Gibbs N., Diamond R., Sekyere E.O., Thomas W.D. (2015). Management of knee osteoarthritis by combined stromal vascular fraction cell therapy, platelet-rich plasma, and musculoskeletal exercises: A case series. J. Pain Res..

