# Supplementary material for: Evaluating the Efficacy of Adipose-Derived Stromal Vascular Fraction Injection for Early-Stage Knee Osteoarthritis: A Multicenter Study
Source: J Clin Med. 2026 May 17;15(10):3855. doi: 10.3390/jcm15103855 (PMC13207802; doi:10.3390/jcm15103855)
Supplement: Supplementary file 1 [file jcm-15-03855-s001.zip › jcm-4284816-supplementary.pdf]

# Supplementary Tables

**Supplementary Table 1:** Post hoc test of VAS scores for Group T

| Post hoc test for Group T | | Statistics | p-value |
|---|---|---|---|
| Preoperative VAS | 1st month VAS | 4,58 | <0.001 |
| Preoperative VAS | 3th month VAS | 5,65 | <0.001 |
| Preoperative VAS | 6th month VAS | 6,82 | <0.001 |
| Preoperative VAS | 12th month VAS | 10,93 | <0.001 |
| 1st month VAS | 3th month VAS | 1,07 | 0.288 |
| 1st month VAS | 6th month VAS | 2,24 | 0.027 |
| 1st month VAS | 12th month VAS | 6,34 | <0.001 |
| 3th month VAS | 6th month VAS | 1,17 | 0.243 |
| 3th month VAS | 12th month VAS | 5,28 | <0.001 |
| 6th month VAS | 12th month VAS | 4,1 | <0.001 |

**Supplementary Table 2:** Post hoc test of VAS scores for Group C

| Post hoc test for Group C | | Statistics | p-value |
|---|---|---|---|
| Preoperative VAS | 1st month VAS | 4.783 | <0.001 |
| Preoperative VAS | 3th month VAS | 1.889 | 0.062 |
| Preoperative VAS | 6th month VAS | 1.122 | 0.265 |
| Preoperative VAS | 12th month VAS | 2.008 | 0.047 |
| 1st month VAS | 3th month VAS | 2.893 | 0.005 |
| 1st month VAS | 6th month VAS | 5.904 | <0.001 |
| 1st month VAS | 12th month VAS | 6.790 | <0.001 |
| 3th month VAS | 6th month VAS | 3.001 | 0.003 |
| 3th month VAS | 12th month VAS | 3.897 | <0.001 |
| 6th month VAS | 12th month VAS | 0.886 | 0.378 |

**Supplementary Table 3:** Post hoc test of WOMAC scores for Group T

| **Post hoc test for Group T** | | **Statistics** | **p-value** |
|---|---|---|---|
| Preoperative WOMAC | 1st month WOMAC | 2.090 | 0.038 |
| Preoperative WOMAC | 3th month WOMAC | 3.086 | 0.002 |
| Preoperative WOMAC | 6th month WOMAC | 6.669 | <0.001 |
| Preoperative WOMAC | 12th month WOMAC | 9.058 | <0.001 |
| 1st month WOMAC | 3th month WOMAC | 0.995 | 0.321 |
| 1st month WOMAC | 6th month WOMAC | 4.579 | <0.001 |
| 1st month WOMAC | 12th month WOMAC | 6.968 | <0.001 |
| 3th month WOMAC | 6th month WOMAC | 3.583 | <0.001 |
| 3th month WOMAC | 12th month WOMAC | 5.972 | <0.001 |
| 6th month WOMAC | 12th month WOMAC | 2.389 | 0.018 |

**Supplementary Table 4:** Post hoc test of WOMAC scores for Group C

| **Post hoc test for Group C** | | **Statistics** | **p-value** |
|---|---|---|---|
| Preoperative WOMAC | 1st month WOMAC | 4.500 | <0.001 |
| Preoperative WOMAC | 3th month WOMAC | 0.653 | 0.515 |
| Preoperative WOMAC | 6th month WOMAC | 5.154 | <0.001 |
| Preoperative WOMAC | 12th month WOMAC | 7.767 | <0.001 |
| 1st month WOMAC | 3th month WOMAC | 5.154 | <0.001 |
| 1st month WOMAC | 6th month WOMAC | 9.564 | <0.001 |
| 1st month WOMAC | 12th month WOMAC | 12.267 | <0.001 |
| 3th month WOMAC | 6th month WOMAC | 4.500 | <0.001 |
| 3th month WOMAC | 12th month WOMAC | 7.113 | <0.001 |
| 6th month WOMAC | 12th month WOMAC | 2.613 | 0.010 |
